# Peritoneal carcinomatosis from ovarian cancer: chemosensitivity test and tissue markers as predictors of response to chemotherapy

**DOI:** 10.1186/1479-5876-9-94

**Published:** 2011-06-20

**Authors:** Chiara Arienti, Anna Tesei, Giorgio Maria Verdecchia, Massimo Framarini, Salvatore Virzì, Antonio Grassi, Emanuela Scarpi, Livia Turci, Rosella Silvestrini, Dino Amadori, Wainer Zoli

**Affiliations:** 1 Biosciences Laboratory, Istituto Scientifico Romagnolo per lo Studio e la Cura dei Tumori (I.R.S.T.), Meldola, Italy; 2 Department of Surgery and Advanced Cancer Therapies, Morgagni-Pierantoni Hospital, Forlì, Italy; 3 Department of Surgery, Bentivoglio Hospital, Bologna, Italy

## Abstract

**Background:**

Platinum-based regimens are the treatments of choice in ovarian cancer, which remains the leading cause of death from gynecological malignancies in the Western world. The aim of the present study was to compare the advantages and limits of a conventional chemosensitivity test with those of new biomolecular markers in predicting response to platinum regimens in a series of patients with peritoneal carcinomatosis from ovarian cancer.

**Methods:**

Fresh surgical biopsy specimens were obtained from 30 patients with primary or recurrent peritoneal carcinomatosis from ovarian cancer. *ERCC1, GSTP1, MGMT, XPD*, and *BRCA1 *gene expression levels were determined by Real-Time RT-PCR. An *in vitro *chemosensitivity test was used to define a sensitivity or resistance profile to the drugs used to treat each patient.

**Results:**

*MGMT *and *XPD *expression was directly and significantly related to resistance to platinum-containing treatment (p = 0.036 and p = 0.043, respectively). Significant predictivity in terms of sensitivity and resistance was observed for *MGMT *expression (75.0% and 72.5%, respectively; p = 0.03), while high predictivity of resistance (90.9%) but very low predictivity of sensitivity (37.5%) (p = 0.06) were observed for *XPD*. The best overall and significant predictivity was observed for chemosensitivity test results (85.7% sensitivity and 91.3% resistance; p = 0.0003).

**Conclusions:**

The in vitro assay showed a consistency with results observed in vivo in 27 out of the 30 patients analyzed. Sensitivity and resistance profiles of different drugs used in vivo would therefore seem to be better defined by the in vitro chemosensitivity test than by expression levels of markers.

## Background

The selection of a chemotherapy regimen for individual tumors is normally based on histology, clinical characteristics of the patient and retrospective evidence from randomized clinical trials. However, patients with the same tumor histotype, especially in solid malignancies, often respond differently to the same chemotherapy regimen due to intertumor heterogeneity. Despite knowledge of such heterogeneity, chemotherapy is still largely empirically planned, and the acquisition of information for tailored therapy has consequently become a priority in the management of cancer patients today.

Such a goal was intensively pursued in the 1980s by American and European research groups who developed a number of chemosensitivity tests using fresh material from human tumors and based on the determination of cell proliferation (clonogenic potential and 3H-thymidine incorporation) or total cell evaluation (dye exclusion, sulphorhodamine blue, MTT assay and ATP bioluminescence) [1-6]. The results obtained from the different tests were compared and their clinical relevance verified in a number of translational clinical studies [5,7-10]. However, various methodological problems and technical skills required have limited the widespread clinical use of *in vitro *experimental results. With the advent of molecular biology at the end of the nineties, attention moved towards the search for molecular and genetic markers involved in proliferation and DNA repair processes that might be predictive of response to both conventional cytotoxic and target therapy drugs [11].

Platinum or platinum-based regimens are the treatment of choice in ovarian cancers, which remains the leading cause of death from gynecological malignancies in the Western world [12]. The absence of specific symptoms in the early stages of the disease results in the majority of patients being diagnosed when the disease is advanced [13]. Currently, standard primary therapy for advanced disease involves surgical debulking followed by platinum/taxane-based chemotherapy [14]. However, despite initially high response rates, a large proportion of patients often experience peritoneal relapse. Recurrent disease is treated with the same regimen used for first-line chemotherapy (i.e., re-induction therapy) or with second- or third-line regimens.

Resistance to platinum alone or in combination is multifactorial. Several studies have attempted to clarify the mechanisms behind resistance to platinum-based chemotherapy, whether intrinsic, as observed in colorectal, prostate, breast or lung cancer, or acquired during treatment. At present, numerous molecular pathways are known to be involved in drug resistance, especially that of platinum compounds. Among such pathways, increased DNA repair and enhanced drug efflux and/or inactivation play an important role in platinum resistance and may also be instrumental in predicting patient prognosis in a clinical setting [11,15,16].

One of the mechanisms involved in DNA repair is the nucleotide excision repair (NER) system, which recognizes helix-distorting base lesions and is presumed to be one of the determinants of platinum resistance [15]. The role of excision repair cross-complementation group1 (*ERCC1*) in the NER pathway is to incise the DNA strand on the 5' site relative to platinated DNA damage, and its overexpression has been associated with clinical resistance to cisplatin [17,18]. Xeroderma pigmentosum group D (*XPD*) is another of the several genes involved in the NER pathway. In particular, *XPD *opens an approximately 30-baseline DNA segment around the damage. It has also been reported that underexpression of *XPD *in cells with transcription coupled-NER-deficiency results in hypersensitivity to cisplatin [19].

DNA adducts at the O6-position of guanine can be repaired by NER but also by O6 methylguanine-DNA methyltransferase (*MGMT*), which is described as a competitor of the NER mechanisms of repair [20]. Preliminary studies have shown that MGMT-deficient cells are unable to repair damage and are more sensitive to the effect induced by alkylating agents than MGMT-proficient cells [21].

Breast cancer gene 1 (*BRCA1*), an essential component of multiple DNA damage repair pathways, is considered to be a differential modulator of survival for cells treated with cisplatin. Preclinical and clinical studies have reported that high levels of *BRCA1 *are associated with cisplatin chemoresistance [18,22,23].

Acquired resistance to DNA adduct formation induced by platinum compounds may be also a consequence of a reduction in drug accumulation in cells due to drug inactivation and/or enhanced efflux. The glutathione *S*-transferase (GST) makes cisplatin more anionic and more readily exported from cells by the ATP-dependent glutathione *S*-conjugate export (GS-X) pump (*MRP1 *or *MRP2*). Some, but not all, translational studies have suggest that the glutathione metabolic pathway may have a role in acquired drug resistance to platinum drugs [15,24,25].

The aims of the present study were to compare the advantages and limits of a conventional chemosensitivity *in vitro *test with those of potentially interesting biomolecular markers in predicting response to platinum or platinum based regimens, in a series of patients with peritoneal carcinomatosis from ovarian cancer.

## Patients and Methods

### Patients

Thirty-two patients with peritoneal carcinomatosis from primary advanced (7 cases) or recurrent (25 cases) ovarian cancer were recruited for the *in vitro *chemosensitivity assay and for analysis of biomarkers potentially predictive of resistance to platinum compounds. Patients underwent surgical resection at Pierantoni Hospital in Forlì and or at Bentivoglio Hospital in Bologna. Inclusion criteria were histological confirmation of advanced or recurrent ovarian cancer and pre- or a postsurgery chemotherapy based on a platinum compound (carboplatin/taxol or cisplatin/adriamycin or carboplatin/gemcitabine or carboplatin as monochemotherapy). It was not possible to perform the *in vitro *chemosensitivity test in 2 patients due to insufficient material. The remaining 30 patients all had serous tumor subtypes. Median age of patients was 60 ± 13.3 years (range 32-81).

Informed consent was obtained before surgical treatment and patients were required to be accessible for follow-up. The study protocol was approved by the Local Ethics Committee. In order to evaluate the correlation between gene expression or *in vitro *chemosensitivity and clinical response to platinum-containing treatment, patients were subdivided into responders (partial or complete clinical response and stable disease) or non-responders (progressive disease).

### Treatment Evaluation

Clinical response was evaluated by measuring circulating CA125 levels before each treatment cycle. Tumor imaging was performed every three cycles using ultrasonography or CT/MRI scans. The same clinical and instrumental evaluation was carried out every 3 months after the end of treatment.

### Sample Collection

Immediately after surgical resection, tumor specimens were sampled and analyzed (under sterile conditions) by a pathologist to confirm the tumor representativity of the samples. A part of the tissue was then stored in RNA*later^® ^*Tissue Collection (Invitrogen, Carlsbad, CA) at a temperature of +4°C to preserve mRNA integrity, while another part was used immediately for the chemosensitivity test.

### Real-Time RT-PCR Analysis

Total RNA was extracted from fresh surgical biopsies using TRIzol^® ^Reagent within 2 or 3 hours of surgery, in accordance with the manufacturer's instructions (Invitrogen). Reverse transcription (RT) reactions were performed in a 20-μl volume containing 800 ng of total RNA using iScript TM cDNA Synthesis kit (Bio-Rad Laboratories, Hercules, CA) and analyzed by Real Time RT-PCR (MyiQ System, Bio-Rad) to detect the expression of the genes *MGMT, BRCA1, ERCC1, GSTP1*, and *XPD*. Primers for mRNA amplification were designed using Beacon Designer Software (version 4, BioRad) and sequences are listed in Table 1. The standard reaction volume was 25 μl containing 2 μl of cDNA template, 1 × SYBR Green Mix and 5 μM of forward and reverse primers. The mixture was subjected to the following cycling conditions: 95°C for 1 min and 30 s, followed by 40 cycles of amplification for 15 s at 95°C and 30 s at 59°C (for *XPD*) or 60°C (for *MGMT, BRCA1, ERCC1, GSTP1, β_2_-microglobulin*, and hypoxanthine phosphoribosyltransferase (*HPRT*)). The amount of mRNA of each marker was normalized to the endogenous references *β_2_-microglobulin *and *HPRT *using Gene Expression Macro Software (Version 1.1) (BioRad). Commercial RNA control derived from a pool of normal ovarian tissue mRNA was used as calibrator.

**Table 1:** Oligonucleotides used for Real-Time PCR

Gene name	5' to 3' forward primer	5' to 3' reverse primer	Annealing temperature
*MGMT*	tcttcaccatcccgttttcc	attgcctctcattgctcctc	60°C
*BRCA1*	gctcgctgagacttcctg	gataaatccatttctttctgttcc	60°C
*ERCC1*	tcagtcaacaaaacggacagtcag	tccttgggttctttcccagagc	60°C
*GSTP1*	aacatgaggcgggcaag	gttgtagtcagcgaaggag	60°C
*XPD*	aagcaggagggcgagaag	cctcatagaatcggcagtgg	59°C
*HPRT*	agactttgctttccttggtcagg	gtctggcttatatccaacattcg	60°C
*Beta2-microglobulin*	cgctactctctctttctggc	agacacatagcaattcaggaat	60°C

The efficiency of amplification, which never exceeded 5% variability in the different experiments, was used to determine the relative expression of mRNA and was calculated using Gene Expression Macro Software (Version 1.1) (BioRad). The reproducibility of Real-Time PCR results was verified in triplicate, and the coefficient of variation (CV), calculated from the three C_t _values, was always < 1.5%.

### *In vitro *Chemosensitivity Test

A cell suspension was obtained after 4-16 hours of enzymatic digestion of fresh tumor tissue. Cells were counted and plated at a density of 1,000,000 cells/well in 96-well flat-bottomed microtiter plates (100 μl of cell suspension/well). Experiments were run in octuplicate. The optical density of treated and untreated cells was determined at a wavelength of 540 nm using a fluorescence plate reader.

Cells were exposed for 72 hours to 1, 10 and 100 μM of cisplatin or adriamycin; 8, 80 and 800 μM of carboplatin; 4, 40 and 400 μM of gemcitabine; and 0.6, 6 and 60 μM of taxol. Drugs were used at concentrations corresponding to peak plasma levels and were also tested at doses equivalent to one-tenth of and tenfold the peak plasma value. Drug activity was assessed by sulforhodamine B assay according to the method of Skehan et al [4]. PC3 tumor cell line, for which the dose-response curve to the anticancer agents used is known, was used as an internal control in all single experiments performed.

### Statistical Analysis

The relationship between continuous (gene expression) and dichotomous variables was analyzed using a non-parametric ranking statistic (median test) [26]. Spearman's correlation coefficient (r_s_) was used to investigate the correlation between the mRNA expression of different genes, such as *MGMT, BRCA1, ERCC1, GSTP1 *and *XPD*, considered as continuous variables. Receiver operating characteristic (ROC) analysis was performed for both individual markers and their combinations. We considered an algorithm that renders a single composite score using the linear predictor fitted from a binary regression model. This algorithm has been justified to be optimal under the linearity assumption [27,28] that the ROC curve is maximized (i.e., best sensitivity) at every threshold value. The chi-square test was used to compare dichotomous variables.

All statistical analyses were performed with SAS Statistical Software (version 9.1, SAS Institute Inc., Cary, NC). Two-sided p values < 0.05 were considered significant.

## Results

The analysis of the comparison between *in vitro *and clinical results was performed on 30 cases with serous tumors. Fifteen patients obtained complete cytoreduction, 6 had minimal residual disease, 4 had maximum residual disease, and the remaining 5 had unresectable disease. The majority of patients (56%) underwent carboplatin/taxol chemotherapy, 20% received cisplatin/adriamycin, 10% carboplatin as monochemotherapy, and 6% carboplatin/gemcitabine or carboplatin/taxol/gemcitabine (Table 2).

**Table 2:** Tumor and patient characteristics and treatment information of the case series

Characteristics	No. patients
**Cancer**	
Primary	**7**
Recurrent	**23**
**Histological type**	
Serous	**30**
**Results of cytoreduction**	
CC0	**15**
CC1	**6**
CC2	**4**
Unresectable	**5**
**Peritoneal Cancer Index ***(mean and range)*	**22.7 (6-39)**
**Type of treatment**	
Carboplatin/taxol	**17**
Cisplatin/adriamicin	**6**
Carboplatin	**3**
Carboplatin/gemcitabine	**2**
Carboplatin/taxol/gemcitabine	**2**

CC0, complete cytoreduction; CC1, minimal residual disease; CC2, maximum residual disease

### Gene Expression Analysis

Of the 5 genes analyzed, *MGMT *and *XPD *expression was directly and significantly related to resistance to cisplatin-including regimens (p = 0.03 and p = 0.04, respectively) (Table 3). In particular, median expression values of *MGMT *and *XPD *in tumors were about fourfold higher in non-responders than in responders.

**Table 3:** Tumor gene expression to platinum-containing treatment in responders and non-responders

	Median expression values (range)			
**Gene**	**Total patients**	**Responders**	**Non-responders**	**p**

*MGMT*	**0.90 **(0-20.0)	**0.57 **(0-2.2)	**2.0 **(0-20.0)	0.03
*XPD*	**0.80 **(0.027-12.4)	**0.52 **(0.027-2.0)	**1.9 **(0.11-12.4)	0.04
*BRCA1*	**2.60 **(0-87.4)	**1.73 **(0.20-6.47)	**3.0 **(0-87.4)	0.59
*ERCC1*	**1.50 **(0.47-15.0)	**2.30 **(0.7-7.02)	**1.4 **(0.47-15.0)	0.93
*GSTP1*	**1.75 **(0.15-45.0)	**1.47 **(0.15-7.5)	**1.7 **(0.71-45.0)	0.65

All 5 genes were generally poorly correlated with each other; with correlation coefficients (r_s_) ranging from 0.577 to 0.074. In particular, of the two genes whose expression was maximally predictive of sensitivity or resistance to clinical treatment, *XPD *was not significantly related to *ERCC1 *or *GSTP1*, and showed borderline clinical significance with *MGMT*. The second, *MGMT*, was significantly related, albeit with a very poor correlation coefficient, to the other four genes (Table 4). The accuracy in predicting sensitivity or resistance to clinical treatment was analyzed for each single gene and for combinations of genes not significantly correlated with each other. Results were expressed as the area under the curve (AUC) and in terms of sensitivity, specificity and overall accuracy (Table 5). AUC values were maximum for *MGMT *(0.73; 95% CI 0.53-0.94) and *XPD *(0.70; 95% CI 0.48-0.91), and different gene combinations did not provide more accurate information. Only the 5 markers considered together slightly improved the AUC value (0.79; CI 0.62-0.97).

**Table 4:** Correlation between *XPD *or *MGMT *and other marker expression

	*XPD*		*BRCA1*		*ERCC1*		*GSTP1*	
	**r_s_**	**p**	**r_s_**	**p**	**r_s_**	**p**	**r_s_**	**p**

***XPD***			0.476	*0.007*	0.074	*0.696*	0.307	*0.099*
***MGMT***	0.355	*0.054*	0.548	*0.002*	0.432	*0.017*	0.577	*0.001*

r_s_, correlation coefficient

**Table 5:** Sensitivity and specificity of individual markers or their combination in predicting response to treatment

	AUC	Cut-off ≥	Sensitivity (%)	Specificity (%)	Overall accuracy (%)
*MGMT*	0.73	0.72	78.9	77.8	78.5
*XPD*	0.70	0.22	89.4	44.4	75.0
*BRCA1*	0.62	2.43	63.1	66.6	64.3
*ERCC1*	0.56	1.37	73.7	44.4	64.3
*GSTP1*	0.57	1.09	63.1	55.5	60.7
*MGMT + XPD*	0.67	-	63.1	55.5	60.7
*XPD + ERCC1*	0.69	-	73.9	44.4	67.8
*XPD + GSTP1*	0.69	-	78.9	44.4	67.8

Five markers together	0.79	-	74.0	77.8	75.0

AUC, area under the curve

These results were paralleled by those expressed as overall accuracy: 78.5% and 75% for *MGMT *and *XPD*, respectively and 75% for the 5 markers considered together. *XPD *expression was characterized by the highest sensitivity (89.4%) but very low specificity (44.4%), while *MGMT *showed both high sensitivity (78.9%) and specificity (77.8%).

### *In Vitro *Chemosensitivity Test

In parallel, a molecular profile of chemosensitivity to all the drugs used in the clinical treatment was generated for each tumor. Patients were subdivided into responders (complete or partial clinical response and stable disease), or non-responders (progressive disease), to evaluate the correlation between *in vitro *chemosensitivity assay and clinical response to platinum-containing treatments (Table 6). Seventeen patients (56.6%) were treated with carboplatin and taxol, of whom 6 had primary advanced and 11 recurrent ovarian cancer. We did not observe any significant differences in either *in vitro *or clinical sensitivity or resistance between primary and recurrent cancers. Considering the 2 subgroups together, concordance between *in vitro *results and clinical response was observed in 14 cases (3 in terms of sensitivity, 11 in terms of resistance). The 3 cases in whom there was no correspondence between *in vitro *and *in vivo *results were all *in vitro *sensitive to one drug (carboplatin or taxol); two showed clinical progression and one stable disease (Table 6). Similarly, in the subgroup of 6 patients treated with cisplatin and adriamycin, 3 were *in vitro-*sensitive to both drugs and showed a clinical response, while 3 were *in vitro *resistant to both drugs and showed disease progression. Patients treated with carboplatin (3 cases: 1 primary and 2 recurrent), carboplatin and gemcitabine (2 cases), or carboplatin, taxol and gemcitabine (2 cases) were *in vitro *resistant to all the drugs and all had disease progression.

**Table 6:** Correspondence between *in vitro *activity and clinical efficacy in individual tumors

	*In vitro *results	Clinical results
**Primary**	***Carboplatin/taxol***	
	S/S	S
	R/S	S
	R/R	R
	R/R	R
	R/R	R
	R/R	R
	***Carboplatin***	
	R	R
**Recurrent**	***Carboplatin/taxol***	
	S/S	S
	R/S	R
	S/S	S
	S/R	R
	R/R	R
	R/R	R
	R/R	R
	R/R	R
	R/R	R
	R/R	R
	R/R	R
	***Cisplatin/adriamycin***	
	R/R	R
	S/S	S
	S/S	S
	S/S	S
	R/R	R
	R/R	R
	***Carboplatin***	
	R	R
	R	R
	***Carboplatin/gemcitabine***	
	R/R	R
	R/R	R
	***Carboplatin/taxol/gemcitabine***	
	R/R/R	R
	R/R/R	R

S, sensitive; R, resistant

### Comparison between the two *In Vitro *Approaches

Results of the clinical response predictivity of the most relevant markers, considered singly or in combination, and of the *in vitro *chemosensitivity test are shown in Table 7. Significant predictivity in terms of sensitivity and resistance to the different cisplatin-based regimens was observed for *MGMT *expression (75.0% and 72.5%, respectively; p = 0.03), while high predictivity with regard to resistance (90.9%), but very low predictivity in terms of sensitivity (37.5%) (p = 0.06) were observed for *XPD*. The combined analysis of the five markers gave the highest predictivity with regard to resistance but very low predictivity in relation to sensitivity (100% and 33.3%, respectively; p = 0.07).

**Table 7:** Predictivity of clinical response by different biomarkers or *in vitro *chemosensitivity test

	Sensitivity (%)	Resistance (%)	p
**Markers**			
*MGMT*	**75.0**	**72.5**	**0.03**
*XPD*	**37.5**	**90.9**	**0.06**
Five markers	**33.3**	**100**	**0.07**
**Chemosensitivity test**	**85.7**	**91.3**	**0.0003**

The best overall and significant predictivity was observed for the *in vitro *chemosensitivity test results (85.7% sensitivity and 91.3% resistance, p = 0.0003). The markers were not effective in predicting resistance or sensitivity to treatment with platinum when recurrent (23) or primary (7) patients were analyzed. Conversely, the chemosensitivity test maintained a significant ability to predict response to chemotherapy in both series of patients.

## Discussion

Prediction of response to drugs at preclinical level could help physicians to plan more effective tailored therapy for individuals, reduce undesirable drug toxicity and lower the cost of health care. In ovarian cancer, despite the heterogeneity of treatments available for peritoneal carcinomatosis, the majority of patients receive platinum-containing chemotherapy in either first- or second- and third-line settings. The use of the re-induction therapy in peritoneal carcinomatosis underlines the importance of studying these patients in terms of preclinical evaluation for response to platinum-containing treatments in order to avoid inactive treatments caused by acquired resistance.

There is a large body of literature highlighting a number of biomarkers as potential candidates for predicting resistance or sensitivity to treatment [11,17-22,29-33]. In the present study, we investigated the role of potentially interesting biomolecular markers and evaluated the relevance of a conventional *in vitro *chemosensitivity test for predicting clinical response to platinum-based regimens in patients with peritoneal carcinomatosis from ovarian cancer.

Among the markers studied, *MGMT *and *XPD *gene expression proved effective in predicting response to platinum-containing therapy. The *MGMT *gene showed good prediction with regard to both sensitivity and resistance, which, is in contrast to results obtained by Codegoni and coworkers who failed to find any relation between *MGMT *expression, detected by northen blot analysis, and response to platinum-based therapy in patients with primary ovarian cancer [34]. *XPD *expression was strongly correlated with drug resistance but weakly associated with drug sensitivity. These results are in agreement with those of Aloyz and coworkers who observed a relationship between *XPD *overexpression and resistance to alkylating agents in human tumor cell lines [35].

In our study the highest predictivity was observed for the *in vitro *chemosensitivity test used to evaluate drug activity. A strong correlation between *in vitro *results and clinical response was observed in 27 out of the 30 patients analyzed, with a predictivity of 85.7% in terms of sensitivity and of 91.3% in terms of resistance. The important predictive relevance of the *in vitro *chemosensitivity test confirms findings published by other authors on a large number of solid and hematologic tumors [9,36-40].

Evaluation of the two analytical approaches highlights the lower cost and higher accuracy, but also the longer execution time and larger amount of tumor material required by the chemosensitivity test compared to Real-Time PCR determination of biomarkers, which gives rapid results using only a few nanograms of RNA.

## Conclusions

In conclusion, it no longer appears ethical to treat patients with drugs to which resistance can be predicted by preclinical experimental techniques in more than 90% of cases. One solution might therefore be to use tumor material from ovarian carcinomatosis as a model for *in vitro *phase II studies to explore the antitumor activity of conventional and novel drugs, singly or in combination.

## List of abbreviations

NER: nucleotide excision repair; *ERCC1*: excision repair cross-complementation group1; *XPD*: xeroderma pigmentosum group D; *MGMT*: O6 methylguanine-DNA methyltransferase; *BRCA1*: breast cancer gene 1; *GST*: glutathione *S*-transferase; RT: reverse transcription; ROC: receiving operating characteristic; AUC: area under the curve.

## Competing interests

The authors declare that they have no competing interests.

## Authors' contributions

WZ, RS, AT and DA designed the study. CA was responsible for data acquisition and carried out the molecular genetic assays and *in vitro *analyses. LT performed the *in vitro *analyses. GMV, MF, SV and AG were responsible for patient recruitment and provided the surgical material. ES performed the statistical analyses. CA, WZ and RS drafted the manuscript. DA and RS reviewed the text for conceptual and analytic integrity. All authors read and approved the final manuscript.
